# Variability in ambient ozone and fine particle concentrations and population susceptibility among Canadian health regions

**DOI:** 10.17269/s41997-018-0169-8

**Published:** 2019-01-07

**Authors:** David M. Stieb, Jiayun Yao, Sarah B. Henderson, Lauren Pinault, Marc H. Smith-Doiron, Alain Robichaud, Aaron van Donkelaar, Randall V. Martin, Richard Ménard, Jeffrey R. Brook

**Affiliations:** 1 Health Canada, Environmental Health Science and Research Bureau, Federal Tower, 420-747 West Hastings St., Vancouver, BC V6C 1A1 Canada; 2 School of Epidemiology and Public Health, University of Ottawa, Room 101 600 Peter Morand Crescent, Ottawa, ON K1G 5Z3 Canada; 3 British Columbia Centre for Disease Control, 655 W 12th Ave, Vancouver, BC V5Z 4R4 Canada; 4 School of Population and Public Health, University of British Columbia, 2206 E Mall, Vancouver, BC V6T 1Z3 Canada; 5 Analytical Studies Branch, Statistics Canada, 100 Tunney’s Pasture Driveway, Ottawa, ON K1A 0T6 Canada; 6 Air Quality Research Division, Environment and Climate Change Canada, 2121, route Transcanadienne, Dorval, QC H9P 1J3 Canada; 7 Department of Physics and Atmospheric Science, Dalhousie University, 6310 Coburg Road, PO Box 15000, Halifax, NS B3H 4R2 Canada; 8 Air Quality Research Division, Environment and Climate Change Canada, 4905 Dufferin St, 4th Floor, Office 4S310, Toronto, ON M3H 5T4 Canada; 9 Dalla Lana School of Public Health, University of Toronto, Health Sciences Building 155 College Street, 6th Floor, Toronto, ON M5T 3M7 Canada

**Keywords:** Air pollution, Ozone, Fine particulate matter, Health effects, Susceptibility, Pollution de l’air, Ozone, Particules fines, Effets sur la santé, Susceptibilité

## Abstract

**Objectives:**

To estimate the proportion of the Canadian population that is more susceptible to adverse effects of ozone (O_3_) and fine particle (PM_2.5_) air pollution exposure and how this varies by health region alongside ambient concentrations of O_3_ and PM_2.5_.

**Methods:**

Using data from the census, the Canadian Community Health Survey, vital statistics and published literature, we generated cross-sectional estimates for 2014 of the proportions of the Canadian population considered more susceptible due to age, chronic disease, pregnancy, outdoor work, socio-economic status, and diet. We also estimated 2010–2012 average concentrations of O_3_ and PM_2.5_. Analyses were conducted nationally and for 110 health regions.

**Results:**

Restrictive criteria (age < 10 or ≥ 75; asthma, chronic obstructive pulmonary disease, heart disease, or diabetes; pregnancy) suggested that approximately one third of the Canadian population is more susceptible, while inclusive criteria (restrictive plus age 10–19 and 65–74, outdoor work, less than high school education, low vitamin C intake) increased this proportion to approximately two thirds. Across health regions, estimates ranged from 24.4% to 41.2% (restrictive) and 61.2% to 87.0% (inclusive). Ten health regions were in the highest quartile of both population susceptibility and O_3_ or PM_2.5_ concentrations, all of which were outside major urban centres.

**Conclusions:**

A substantial proportion of the Canadian population exhibits at least one risk factor that increases their susceptibility to adverse effects of O_3_ and PM_2.5_ exposure. Both risk communication and management interventions need to be increasingly targeted to regions outside large urban centres in the highest quartiles of both susceptibility and exposure.

**Electronic supplementary material:**

The online version of this article (10.17269/s41997-018-0169-8) contains supplementary material, which is available to authorized users.

## Résumé

**Objectifs:**

Estimer la proportion de la population canadienne qui est plus susceptible aux effets indésirables de l’exposition à la pollution de l’air par l’ozone (O_3_) et les particules fines (PM_2,5_), ainsi que les variations de cette proportion selon la région sanitaire et les concentrations ambiantes d’O_3_ et de PM_2,5_.

**Méthode:**

À l’aide des données du recensement, de l’Enquête sur la santé dans les collectivités canadiennes, des statistiques de l’état civil et des travaux publiés, nous avons généré des estimations transversales, pour l’année 2014, des proportions de la population canadienne jugées plus susceptibles aux effets de la pollution de l’air en raison de l’âge, des maladies chroniques, de la grossesse, du travail en plein air, du statut socioéconomique et du régime alimentaire. Nous avons aussi estimé les concentrations moyennes d’O_3_ et de PM_2,5_ en 2010-2012. Des analyses ont été menées pour l’ensemble du pays et pour 110 régions sanitaires.

**Résultats:**

Selon les critères restrictifs (< 10 ans ou ≥ 75 ans; asthme, maladie pulmonaire obstructive chronique, cardiopathie ou diabète; grossesse), environ le tiers de la population canadienne est plus susceptible aux effets de la pollution de l’air, tandis que les critères inclusifs (critères restrictifs plus : 10 à 19 ans et 65 à 74 ans; travail en plein air; niveau de scolarité inférieur au diplôme d’études secondaires; faible apport en vitamine C) gonflent cette proportion à environ les deux tiers. Dans les régions sanitaires, les estimations ont varié entre 24,4 % et 41,2 % (critères restrictifs) et entre 61,2 % et 87 % (critères inclusifs). Dix régions sanitaires se classaient dans le quartile supérieur tant pour la susceptibilité de leur population que pour les concentrations d’O_3_ ou de PM_2,5_; toutes étaient situées hors des grands centres urbains.

**Conclusions:**

Une proportion considérable de la population canadienne présente au moins un facteur de risque qui augmente sa susceptibilité aux effets indésirables de l’exposition à l’O_3_ et aux PM_2,5_. La communication des risques et les interventions de gestion doivent être de plus en plus ciblées sur les régions hors des grands centres urbains qui se classent dans les quartiles supérieurs pour la susceptibilité et l’exposition.

## Introduction

There is a large and continually growing evidence base linking both short- and long-term air pollution exposures to acute and chronic health impacts. These include well-established respiratory and cardiovascular effects, and, more recently, effects on metabolic function, pregnancy, developmental, neurologic, and psychiatric outcomes (Thurston et al. 2017). Ozone (O_3_) and fine particulate matter (PM_2.5_) have been of particular interest in Canada and elsewhere because of their well-documented adverse health effects (Thurston et al. 2017), as well as their tendency to affect large airsheds in the form of photochemical smog resulting from atmospheric processes (Brook et al. 2014). Elevated concentrations of both O_3_ and PM_2.5_ in Canada result from emissions of precursor pollutants (sulfur dioxide, nitrogen oxides, and volatile organic compounds) from mobile and stationary sources, as well as long-range transport of these pollutants and their precursors, particularly from the United States (Brook et al. 2014). Concentrations of O_3_ are generally higher during summer months, while elevated PM_2.5_ concentrations may occur in winter months in areas where residential wood heating is prevalent, compounded by temperature inversions and valley topography that trap pollutants (Brook et al. 2014).

While the entire population can be exposed to ambient air pollution, certain subpopulations appear to be at higher risk of experiencing significant health effects from acute or chronic exposures. These groups have been referred to inconsistently in the literature as demonstrating greater “sensitivity,” “susceptibility,” or “vulnerability.” While no single term is inherently superior, here, we have chosen “susceptibility” in keeping with recent reviews (Vinikoor-Imler et al. 2014; Sacks et al. 2011). We interpret susceptibility as a gradient rather than as a dichotomous quality. Further, individuals without recognized risk factors for susceptibility may still experience adverse effects from air pollution exposure. Chronic disease, especially heart disease, asthma, chronic obstructive pulmonary disease (COPD), and diabetes, as well as older age confer greater susceptibility related to impaired physiological function (Vinikoor-Imler et al. 2014; Sacks et al. 2011; Johnson and Graham 2005). Younger age confers greater susceptibility because children’s lungs continue to develop until at least 18 years of age, children inhale more air per kilogram body weight, and they tend to be more physically active outdoors (Vinikoor-Imler et al. 2014; Sacks et al. 2011; Johnson and Graham 2005). Unborn children are also at risk, as several studies have reported that air pollution exposure is associated with reduced fetal growth and preterm birth, including work conducted in Canada (Stieb et al. 2016). Other studies suggest that people with low socio-economic status (SES) are also at greater risk because of reduced access to healthcare and nutritious food, and increased prevalence of chronic disease, and that low intake of vitamins C and E impairs defenses against oxidative stress (Vinikoor-Imler et al. 2014; Sacks et al. 2011). Finally, outdoor workers are at increased risk because of their proximity to exposure, and increased ventilation rate during strenuous work (Vinikoor-Imler et al. 2014).

Few studies have attempted to estimate the size of the more susceptible population, which is needed to ensure that these populations are accurately identified and appropriately targeted by risk communication and management activities. Here, we have estimated the proportion of the Canadian population that falls into at least one of these more susceptible subpopulations, and how this varies throughout the country alongside long-term exposure to O_3_ and PM_2.5_.

## Methods

### Data on risk factor prevalence

We generated cross-sectional estimates of the proportions of the 2014 Canadian population in the following categories: (1) children and seniors; (2) individuals with heart disease, asthma, COPD, or diabetes; (3) pregnant women; (4) outdoor workers; (5) adults with less than a high school education, as an indicator of low SES; and (6) individuals with low vitamin C intake as an indicator of impaired defenses (Table 1). Data on the population size and age distribution were obtained from Statistics Canada estimates (Statistics Canada 2016a). The proportion of pregnant women in the population at any given time was calculated using data from Statistics Canada (Statistics Canada 2016b) and the Canadian Institute for Health Information (Canadian Institute for Health Information 2014), using methods described elsewhere for emergency preparedness (U.S. Centers for Disease Control 2016). Age-specific estimates of the prevalence of chronic diseases were obtained from combined 2013 and 2014 data from the Canadian Community Health Survey (CCHS), which is an annual national cross-sectional survey of individuals 12 years of age and over (Statistics Canada 2014). The sample size for respondents who agreed to share their data with Health Canada was 121,486 (approximately 93% of all respondents). Estimates of low SES were taken from the same data. Presence of chronic disease was determined by self-report of “*long-term conditions* which are expected to last or have already lasted 6 months or more and that have been diagnosed by a health professional.” Age-stratified values for the prevalence of low vitamin C intake were based on an analysis of CCHS 24-h dietary recall data, standard reference data on the nutrient composition of foods, and data on supplement consumption (Shakur et al. 2012). Prevalence of outdoor work stratified by age was based on a Canadian national survey on sun exposure and protective behaviours (Marrett et al. 2010) and was further stratified by urban vs. rural place of residence based on results from a national time-activity patterns survey (Matz et al. 2015).

**Table 1:** Definitions of more susceptible subpopulations by approach and sources of data

Susceptibility factor	Approach		Year	Geographic resolution	Source
	Restrictive	Inclusive			
Age (children)	< 10 years old	< 20 years old	2014	Health region	Statistics Canada population estimates (Statistics Canada 2016a)
Age (seniors)	≥ 75 years old	≥ 65 years old			
Chronic disease	Individuals with heart disease, asthma, chronic obstructive pulmonary disease, or diabetes		2013, 2014	Health region	Statistics Canada Canadian Community Health Survey (Statistics Canada 2014)
Pregnancy	Pregnant women		2013	Province	Statistics Canada fertility rates (Statistics Canada 2016b)
			2005		Statistics Canada fetal loss rates (Statistics Canada 2016b)
			2014		Canadian Institute for Health Information induced abortion data (Canadian Institute for Health Information 2014)
Outdoor work		Outdoor workers	2006	National (adjusted by age group and urban/rural status by health region)	(Marrett et al. 2010)
			2010, 2011		(Matz et al. 2015)
Socio-economic status		Less than high school education	2013, 2014	Health region	Statistics Canada Canadian Community Health Survey (Statistics Canada 2014)
Impaired defenses		Low vitamin C intake	2004	National (adjusted by age group by health region)	(Shakur et al. 2012)

### Estimation of the national and regional susceptible subpopulations

**Fig. 1:**
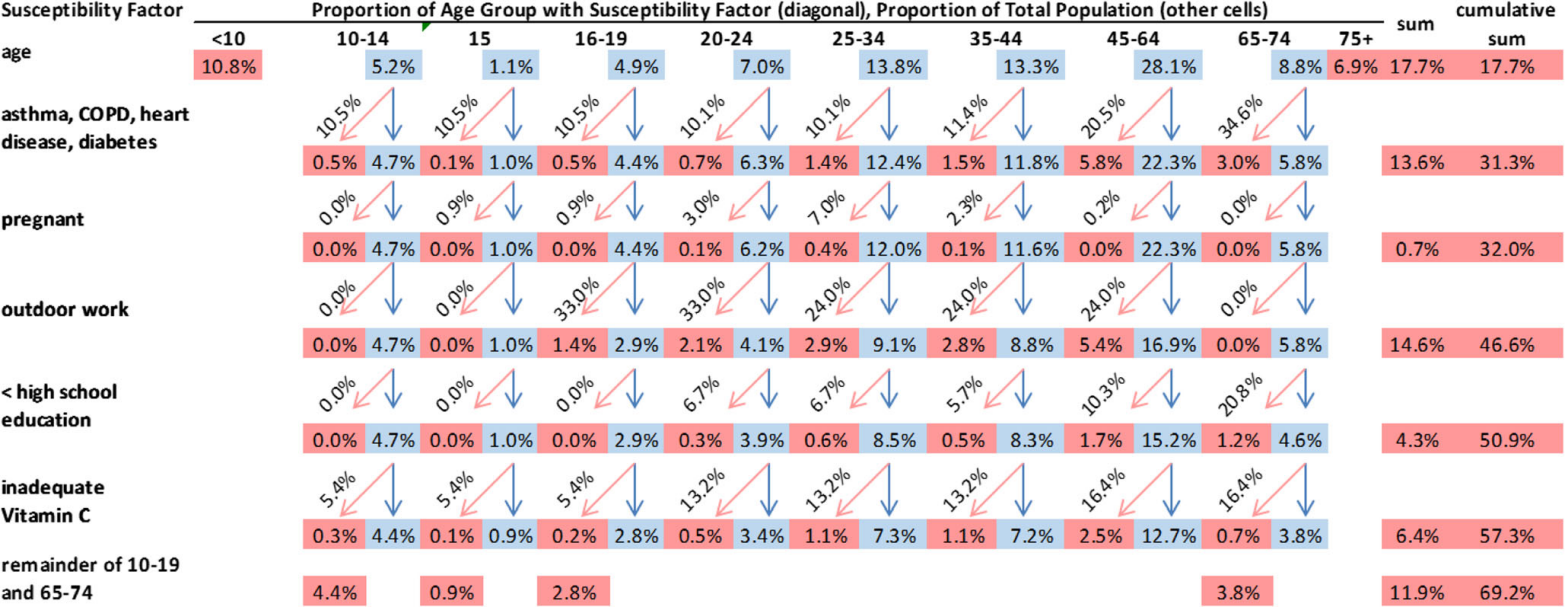
Illustration of the calculation of the percent of the population classified as more susceptible. The percentages in pink are the proportions of the population at risk for each risk factor (rows) in each age category (columns). The percentages in blue are the proportions of the population not at risk, which are carried through to the next risk factor. The diagonal percentages (pink arrows) indicate the prevalence of the next risk factor for the specific age group that is applied to the population not at risk from the previous risk factors. Percent pregnant is applied to the female proportion of the population in each age group. Rows and columns may not sum to marginal totals due to rounding

To account for the breakdown of other susceptibility factors by age, the population was divided into ten categories as follows: < 10, 10–14, 15, 16–19, 20–24, 25–34, 35–44, 45–64, 65–74, and ≥ 75 years (the 10–19 age group was subdivided into groups of unequal length because estimation of the prevalence of pregnancy included 15-year-olds, while estimation of the prevalence of outdoor work began with 16-year-olds). The population at risk within the age category was then assessed for the other risk factors in sequence, starting with chronic disease and ending with inadequate vitamin C intake. At every step, the proportion of the population remaining within each age category was multiplied by the prevalence of the next risk factor for that age category, and the resulting proportion at risk was removed from further calculations (Fig. 1). The total percent of the population classified as more susceptible was the sum of the percent of the population with at least one risk factor, assuming independence between the risk factors where data on dependence were not available.

Calculations were conducted using two approaches: restrictive and inclusive (Table 1). We chose to contrast these approaches to examine the sensitivity of estimates to the types and number of risk factors considered. Analyses were conducted nationally and for 110 administrative health regions defined by provincial ministries of health. In some jurisdictions, health regions correspond to areas served by local public health departments or authorities. The proportion of the population residing in urban vs. rural areas within each health region was determined using CCHS data classifying respondent place of residence based on census geography. Statistics Canada classifies areas as (urban) population centres if the population is 1000 or more and population density is 400 or more per square kilometre; large population centres are those with a population of 100,000 or more (Statistics Canada 2016c). The CCHS is designed to be representative at the health region scale. Analysis of CCHS data accounted for the complex sampling design using survey weights, employing the Descr package (Aquino 2016) in R version 3.3.1 (R Core Team 2016).

### Estimation of exposure to O_3_ and PM_2.5_

While identifying areas with a higher proportion of more susceptible individuals is of interest, it is especially important to identify populations that are both more highly susceptible and more highly exposed. We overlaid the proportion of the population estimated to be more susceptible in each health region with estimated 2010–2012 (the most recent years available) average concentrations of O_3_ and PM_2.5_, derived from published model-observation fusion techniques (Robichaud and Ménard 2013; van Donkelaar et al. 2015). The 3-year average is considered stable relative to potentially anomalous single-year results. These estimates were drawn from multi-year surfaces produced as inputs for epidemiological studies (see Online Resource 1). We employed continuous pollutant concentrations rather than examining compliance with standards because standards are not strictly health-based (there are no known thresholds for O_3_ and PM_2.5_ below which adverse effects on health do not occur), but rather are management targets which attempt to balance health protection and cost of emission controls (Canadian Council of Ministers of the Environment 2007). We divided health regions into quartiles based on the proportion of the population identified as more susceptible according to restrictive and inclusive criteria, as well as concentrations of O_3_ and PM_2.5_. As such, each health region was classified into one of 16 susceptibility/pollutant concentration categories (ranging from low/low to high/high) for each susceptibility calculation and pollutant.

## Results

### National susceptibility

The restrictive approach suggested that approximately one third (32.0%) of the Canadian population is more susceptible to air pollution, while the inclusive approach suggested that approximately two thirds of the population (69.2%) is more susceptible (Fig. 1). The restrictive approach was driven by the proportions of children and the elderly, and of individuals with chronic diseases; the prevalence of pregnancy was comparatively much lower. The inclusive approach was also impacted by the estimated percentage of outdoor workers (33% for young adults and 24% for older adults) and of those without chronic disease in the 10–19 and 65–74-year age groups. The impact of low SES was less prominent due to its generally lower prevalence in the population, ranging from 7% to 21% for different age groups.

### Regional susceptibility

**Fig. 2:**
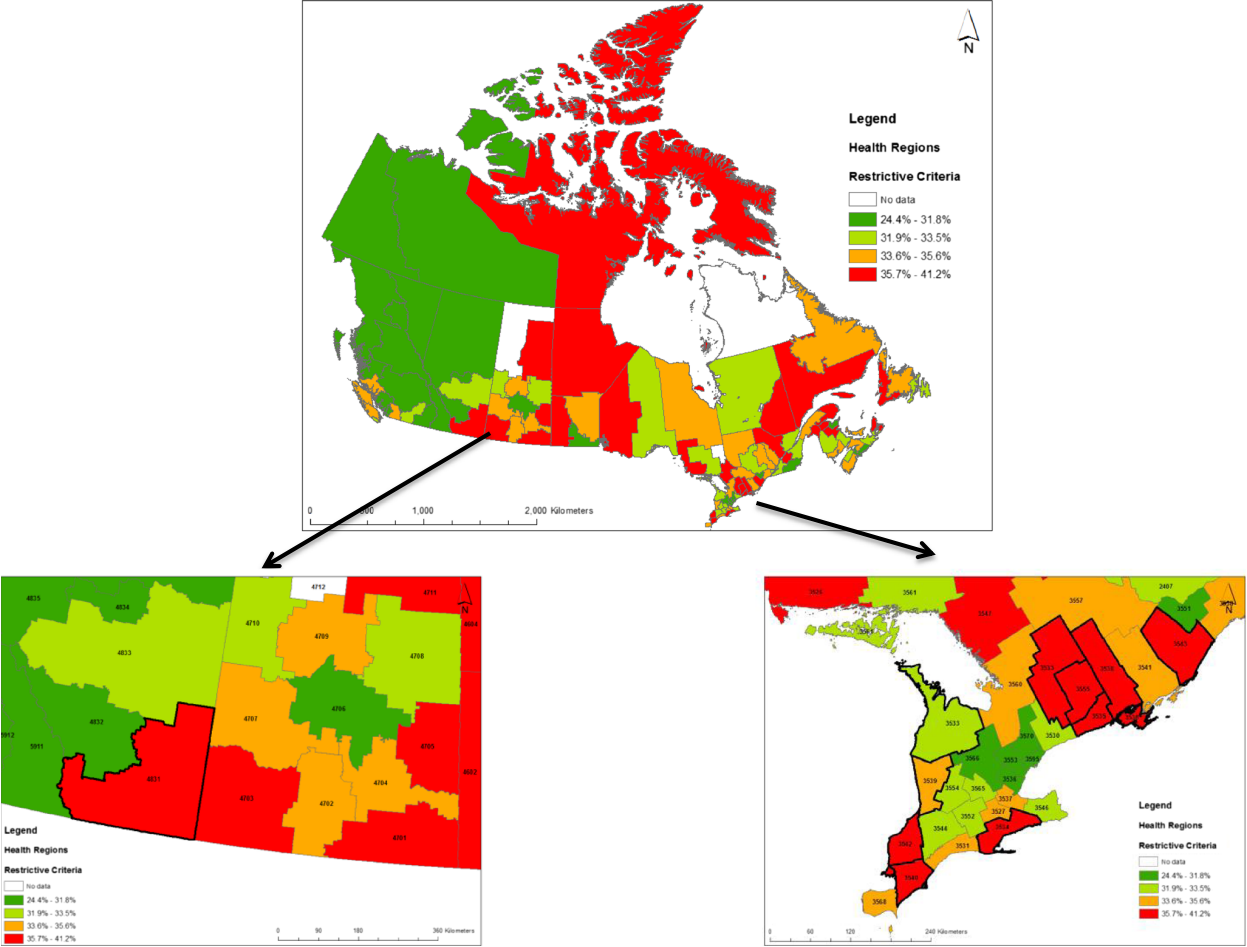
Percent of population classified as more susceptible to adverse effects of air pollution by health region according to restrictive criteria. Insets show detail for southern Alberta and southern Ontario; health regions outlined in bold are in the highest quartile of percent susceptible according to restrictive and/or inclusive criteria and highest quartile of PM_2.5_ and/or O_3_

Across the 110 health regions, estimates of the more susceptible population ranged from 24.4% to 41.2% using the restrictive approach (Table 2 and Fig. 2) and from 61.2% to 87.0% using the inclusive approach (Table 2, Online Resource 2). The percent of the population classified as more susceptible according to restrictive and inclusive criteria was highly correlated (Spearman *ρ* = 0.77, *p* < 0.0001). The most susceptible populations were generally located outside of large urban centres, and the percent classified as more susceptible according to inclusive criteria was highly correlated with the percent of the population considered rural (Spearman *ρ* = 0.81, *p* < 0.0001). Population age distribution and prevalence of chronic disease and less than high school education exhibited the greatest variability among health regions (Table 2). Results for individual health regions and a calculator tool are provided in Online Resources 3 and 4, respectively.

**Table 2:** Variability of prevalence (%) of susceptibility factors by health region (*n* = 110)

	Number or percent of health region population					
Characteristic	Minimum	25th percentile	Median	Mean	75th percentile	Maximum
Population (*n*)	14,153	82,400	164,616	322,662	417,167	2,804,607
Urban (%)	10.4	56.3	67.3	67.5	82.6	100.0
Rural (%)	0.0	17.4	32.8	32.6	43.8	89.6
1. Age < 10 (%)	7.3	9.6	10.6	11.0	11.6	22.3
2. Age 75+ (%)	0.9	6.1	7.4	7.2	8.6	10.9
3. Asthma, COPD, heart disease, diabetes (age 10–74) (%)	8.8	12.8	14.8	14.7	16.6	22.4
4. Pregnant (%)	0.5	0.6	0.7	0.7	0.8	1.8
5. Outdoor work (%)	13.0	14.3	14.9	15.1	15.9	18.4
6. Less than high-school education (%)	1.4	4.0	4.8	5.3	6.3	18.2
7. Inadequate vitamin C intake (%)	2.9	5.6	5.9	6.0	6.4	7.7
8. Remainder of 10–19 and 65–74 (%)	9.8	11.2	12.1	12.1	13.1	15.1
Restrictive (sum of factors 1–4) (%)	24.4	31.8	33.5	33.6	35.6	41.2
Inclusive (sum of factors 1–8) (%)	61.2	69.8	72.3	72.1	74.4	87.0

### Susceptibility vs. ambient concentration

**Fig. 3:**
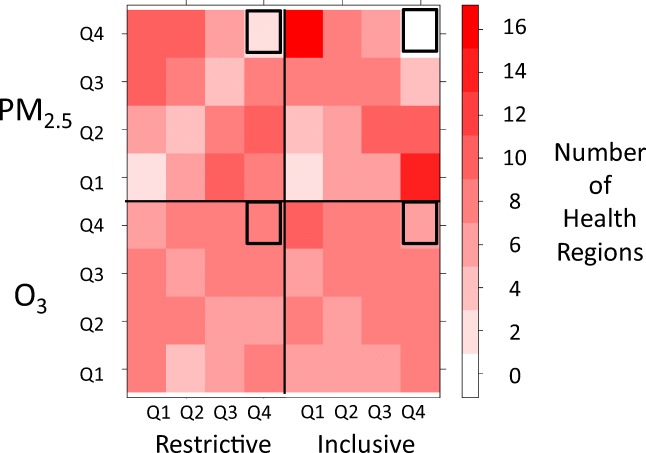
Heat map of quartiles of percent susceptibility according to restrictive and inclusive criteria vs. quartiles of PM_2.5_ and O_3_ for 110 health regions. Bold outline highlights correspondence of highest quartiles of both susceptibility and exposure (see also insets of Fig. 2 and Online Resource 2)

A heat map of quartiles of susceptibility and long-term PM_2.5_ and O_3_ concentrations by health region is provided in Fig. 3 (see also Online Resource 5 for underlying data). Health regions in the lower quartiles of susceptibility according to both restrictive and inclusive criteria tended to be in the highest quartiles of PM_2.5_ concentrations, while those in the highest quartiles of susceptibility tended to be in the lowest quartiles of PM_2.5_ concentrations. In contrast, the distribution of susceptibility quartiles across pollutant concentration quartiles was relatively balanced for O_3_. PM_2.5_ concentrations were negatively correlated with percent of the population classified as more susceptible according to both restrictive (Spearman *ρ* = − 0.33, *p* = 0.0005) and inclusive criteria (Spearman *ρ* = − 0.57, *p* < 0.0001), while O_3_ concentrations were uncorrelated with percent classified as more susceptible (Spearman *ρ* = 0.11, *p* = 0.25 and *ρ* = − 0.07, *p* = 0.50, respectively). Further, PM_2.5_ concentrations were also correlated with the percent of the health region population considered urban (Spearman *ρ* = 0.65, *p* < 0.0001) while O_3_ concentrations were not (Spearman *ρ* = 0.14, *p* = 0.15). We identified ten health regions in the highest quartile of population susceptibility according to either restrictive or inclusive criteria which were also in the highest quartile of O_3_ or PM_2.5_ concentrations (Fig. 2 and Online Resources 2 and 6). Of these, nine were in southern Ontario and one was in southern Alberta; all were outside major urban centres and seven consisted of at least 40% rural population. All ten were in the highest quartile of O_3_ concentrations and two were also in the highest quartile of PM_2.5_ concentrations. Most exceeded the median for the prevalence of population ≥ 75 (all 10 regions), chronic disease (8 of 10 regions), or population 10–19 or 65–74 without chronic disease (7 of 10 regions). We evaluated the stability of PM_2.5_ and O_3_ concentrations by also calculating 10-year averages (2003–2012). The 10 regions remained in the highest quartile of 10-year average O_3_ concentrations, and three of these were also in the highest quartile of 10-year average PM_2.5_ concentrations. The correlation between 10-year average values and 3-year average values was very high (*R*^2^ of 0.92 and 0.96, respectively for PM_2.5_ and O_3_).

## Discussion

Our findings indicate that a substantial proportion of the Canadian population exhibits at least one risk factor that increases susceptibility to the adverse effects of air pollution exposure, even employing restrictive criteria. Applying more inclusive criteria results in a substantial majority of the population having at least one risk factor. Age, presence of chronic disease, and outdoor work were the most common risk factors, while age, chronic disease, and low SES had the greatest variability among health regions. Johnson and Graham (2005) estimated a somewhat higher proportion of those < 18 and 65+ (38% vs. 30% in our analysis) in the eastern seaboard states of CT, ME, MA, NH, NJ, NY, RI, and VT (Johnson and Graham 2005). They also used data based on self-report from health surveys to estimate the prevalence of the same chronic diseases, but did not account for the joint presence of multiple conditions. They relied on ground-based monitoring data to estimate the proportion of the overall and more susceptible populations who would benefit from achievement of alternative air quality standards.

We found that more susceptible populations coincided with higher O_3_ and PM_2.5_ concentrations only in health regions located outside of large urban centres, and primarily in health regions where at least 40% of the population was classified as rural. Of the susceptibility factors we considered (age, chronic disease, pregnancy, outdoor work, socio-economic status, and diet), only chronic disease could plausibly occur as a consequence of air pollution exposure. In addition to factors we considered, it has also been reported that rural populations spend more time outdoors, are more likely to work in strenuous outdoor occupations and are less likely to have air conditioning, all of which would increase exposure to air pollution (Matz et al. 2015). Greater time outdoors, particularly in relation to leisure time physical activity, is likely to confer health benefits, but occupational physical activity may have primarily negative health impacts (Holtermann et al. 2012). Although there is considerable heterogeneity among rural areas, in general, rural populations tend to be older (Statistics Canada 2018) and in poorer health (Pinault et al. 2016) than urban populations. This may be partly attributable to migration of younger adults from rural to urban areas (Statistics Canada 2018), and a lack of inflow of comparatively healthier immigrants who tend to settle in urban rather than rural areas (Ng 2011). Environmental justice researchers have applied the term “double jeopardy” to describe the co-occurrence of high exposures and high susceptibility related to socio-economic stressors, primarily in disadvantaged urban areas (Morello-Frosch and Shenassa 2006). Our findings highlight that this concept can also apply to rural areas where higher ambient concentrations related to long-range transport can coincide with increased susceptibility related to age, chronic disease, and outdoor work. To the extent that the population is aging (particularly in rural areas) and ozone concentrations are projected to increase in relation to climate change (Smith et al. 2014), the degree of co-occurrence of high-exposure and high susceptibility in rural areas may increase. While urban areas also experience elevated exposures to O_3_ and PM_2.5_, and tend to be the focus of both risk communication and management for these pollutants, in our analysis, none were also in the highest quartile of percent of the population more highly susceptible. Our findings of the co-occurrence of increased susceptibility and exposure only outside large urban centres, together with evidence of a relative lack of awareness about air pollution in these areas (Environment and Climate Change Canada 2013), suggest that increased attention to both risk communication and risk management pertaining to O_3_ and PM_2.5_ is needed outside large urban centres. Risk communication activities may include increasing awareness among both public health professionals and the general public of communication tools like the Air Quality Health Index (AQHI) (Environment and Climate Change Canada 2008). The AQHI addresses short-term exposure related to hour to hour and day to day changes in air quality, but new risk communication vehicles are needed to address long-term exposure. Specifically targeting at-risk groups in these areas (children, the elderly, those with chronic respiratory or cardiovascular disease or diabetes, pregnant women, individuals with lower SES or poor diet, and outdoor workers) is recommended, but may require innovative approaches to effectively reach more widely dispersed populations. In areas such as these without local sources, elevated concentrations of O_3_ and PM_2.5_ stem primarily from long-range atmospheric transport from upwind urban centres (Brook et al. 2014). In the case of O_3_, there is also a lack of chemical scavenging by oxides of nitrogen, which occurs principally in urban areas with heavy traffic (Brook et al. 2014). Risk management therefore requires attention to national and international agreements addressing long range-transported air pollution as well as a variety of measures to reduce emissions of primary particulate matter as well as precursors of O_3_ and PM_2.5_. These include reduction of emissions from stationary sources such as industrial sectors and power generation, as well as from mobile (transportation) sources through vehicle and fuel standards, and from consumer products (Canadian Council of Ministers of the Environment 2010). Specific local sources such as agricultural burning or residential wood burning may also need to be addressed. Where sources are poorly understood, dispersion modeling or analysis of back trajectories may be needed.

### Strengths and limitations

By accessing national health survey microdata on a representative sample of over 120,000 participants, we were able to directly estimate the prevalence of chronic conditions by age group and health region, accounting for the joint presence of two or more conditions and of chronic conditions and low educational attainment. Prevalence of chronic disease was based on self-report, which may introduce error. In a study comparing self-reported chronic health conditions in 2001, 2003, and 2005 cycles of the CCHS with identification of these conditions using linked administrative data, agreement was good for diabetes (*κ* = 0.8), although prevalence was underestimated by self-report (6.8% vs. 8.4%); moderate for asthma (*κ* = 0.55), for which prevalence was also underestimated slightly (8.6% vs. 9.8%); and low for COPD (*κ* = 0.29), for which prevalence was substantially underestimated (5.6% vs. 11.1%) (Muggah et al. 2013). Heart disease as a general condition was not included in this analysis. However, agreement was high for hypertension (*κ* = 0.66), moderate for myocardial infarction (*κ* = 0.48), and low for congestive heart failure (*κ* = 0.33) (Muggah et al. 2013). Prevalence of hypertension and congestive heart failure was underestimated by self-report, while that of myocardial infarction was overestimated. An earlier Canadian study, also linking health survey and administrative data, found similar levels of agreement for diabetes and hypertension (Robinson et al. 1997). Overall, these studies suggest that our estimates of the prevalence of these conditions may be lower than the true population prevalence. While estimates of the prevalence of inadequate vitamin C intake is based on accepted methods, these estimates were not validated by clinical measures (Shakur et al. 2012). We also did not have estimates of the prevalence of inadequate vitamin E intake.

A key strength of our analysis is the availability of nationally comprehensive air pollution data derived from remote sensing, air quality models, and ground observations. This allowed us to include rural areas without monitoring, which has been a shortcoming of earlier analyses of the co-occurrence of increased susceptibility and pollutant concentrations (Miranda et al. 2011). However, our examination of spatial variability of susceptibility and air pollution was restricted to relatively large areas because data on chronic disease prevalence were not available nationally at a higher resolution. We recognize that chronic disease prevalence is likely to vary at a smaller spatial scale, particularly in dense urban areas. For this reason, we focused on air pollutants (O_3_ and PM_2.5_) that tend to be more homogeneous over larger areas. We also acknowledge that PM_2.5_ may vary at a smaller scale in rural areas in relation to sources such as wood smoke, and O_3_ may be titrated by oxides of nitrogen at a smaller scale in urban areas. In order to stabilize estimates of air pollution concentrations, our analysis was based on 3-year average concentrations and thus would not capture the influence of annual variations in weather or wildfire activity. We also did not account for increased exposure to other pollutants or related to proximity to roads or other sources. Brauer et al. estimated that one third of Canadians live in areas with high levels of exposure to traffic-related air pollution (within 500 m of highways or 100 m of major roads) (Brauer et al. 2013). In contrast to O_3_ and PM_2.5_ which tend to be relatively spatially homogeneous, traffic-related pollutants are found within a short distance of major roads.

We did not account for increased susceptibility related to genetic factors (Vinikoor-Imler et al. 2014; Sacks et al. 2011). In a review of glutathione *S*-transferase M1 (GSTM1) polymorphisms and lung cancer, the median prevalence of the homozygous null genotype was 50% among control subjects in 98 epidemiologic studies (Carlsten et al. 2008). This suggests that even consideration of a single genotype would result in a substantial further increase in the proportion of the population considered more susceptible. While the risk factors we evaluated here probably differ in the extent to which they modify susceptibility, there is insufficient evidence to reliably derive and apply quantitative weights. Nonetheless, we have employed a “restrictive” vs. “inclusive” approach to illustrate the sensitivity of our findings to the number and types of factors considered. There is also likely to be considerable heterogeneity in susceptibility even within the broad population groups considered based on the evidence to be more susceptible. Ongoing research is needed to continue to evaluate factors modifying susceptibility to the full spectrum of air pollutants, to examine heterogeneity in susceptibility within broad population groups, and to characterize geographic variability in susceptibility and exposure.

## Electronic supplementary material

Online Resource 1 (PDF 30 kb)

Online Resource 2 (PPTX 485 kb)

Online Resource 3 (XLSX 36 kb)

Online Resource 4 (XLSX 16 kb)

Online Resource 5 (XLSX 11 kb)

Online Resource 6 (XLSX 19 kb)
