# Supplementary material for: Variability in ambient ozone and fine particle concentrations and population susceptibility among Canadian health regions
Source: Can J Public Health. 2019 Jan 7;110(2):149–58. doi: 10.17269/s41997-018-0169-8 (PMC6964403; doi:10.17269/s41997-018-0169-8)
Supplement: Supplementary file 1 — (PDF 30 kb) [file 41997_2018_169_MOESM1_ESM.pdf]

Online Resource 1, Additional details on exposure surfaces

Stieb DM*, Yao J, Henderson SB, Pinault L, Smith-Doiron MH, Robichaud A, van Donkelaar A, Martin RV, Ménard R, Brook JR. Variability in ambient ozone and fine particle concentrations and population susceptibility among Canadian health regions. Can J Public Health

*corresponding author, Health Canada and School of Epidemiology and Public Health, University of Ottawa, dave.stieb@canada.ca

$PM_{2.5}$ exposures were estimated from the total column aerosol optical depth (AOD) measured by satellite, using relationships between AOD and near-surface $PM_{2.5}$ derived from a chemical transport model.[1] Yearly average surface layers of approximately 1 km^2 resolution were obtained by applying geographically weighted regression for the 2004-2012 period. Estimates of $PM_{2.5}$ over 20 $\mu g/m^3$ were excluded from the analysis because they are believed to be due to inaccurate satellite retrievals.[2]

8-hour daily maximum O_3 exposures were estimated for the months of May to October (the period during which concentrations are generally highest due to O_3's atmospheric chemistry) during the 2002-2009 period using data assimilation techniques (optimum interpolation) to generate a 21 km^2 grid.[2] This approach combines O_3 concentrations from the Canadian Hemispheric Regional O_3 and NOx system (CHRONOS), an operational regional air quality forecast model,[3] and ground-based observations from monitors in Canada and the United States. The O_3 estimates were adjusted for annual differences using a time series of ground measurements from 24 census divisions (CD) over the 1981-2012 period. For each of the 24 CDs, a cubic spline function was fitted to model the association between year and air pollutant. Ratios were determined for each year relative to the source data year. All locations were matched to the closest CD using Geographic Information Systems (GIS; ArcGIS v.10, ESRI 2010), and the corresponding time adjustment ratio was used to adjust data for annual differences in concentration.

Population-weighted pollutant concentration estimates for each health region were calculated using the residential locations of 2011 Census respondents, determined using the residential postal codes reported on the census files and the Postal Code Conversion File plus (PCCF+) v.6c.[4]

References

1. van Donkelaar A, Martin RV, Spurr RJD, et al. High-Resolution Satellite-Derived PM2.5 from Optimal Estimation and Geographically Weighted Regression over North America. *Environ Sci Technol*. 2015;49(17):10482–91.
2. Robichaud A, Ménard R. Multi-year objective analyses of warm season ground-level ozone and PM2.5 over North America using real-time observations and Canadian operational air quality models. *Atmospheric Chem Phys Discuss*. 2013;13:13967–4035.
3. Pudykiewicz JA, Kallaur A, Smolarkiewicz PK. Semi-Lagrangian modelling of tropospheric ozone. *Tellus B Chem Phys Meteorol*. 1997;49(3):231–48.
4. Statistics Canada. *Postal Code Conversion File Plus (PCCF+) Version 6C, Reference Guide Cat no. 82-F0086-XDB.* 2016.
