# Supplementary material for: Variability in ambient ozone and fine particle concentrations and population susceptibility among Canadian health regions
Source: Can J Public Health. 2019 Jan 7;110(2):149–58. doi: 10.17269/s41997-018-0169-8 (PMC6964403; doi:10.17269/s41997-018-0169-8)

## Slide 1
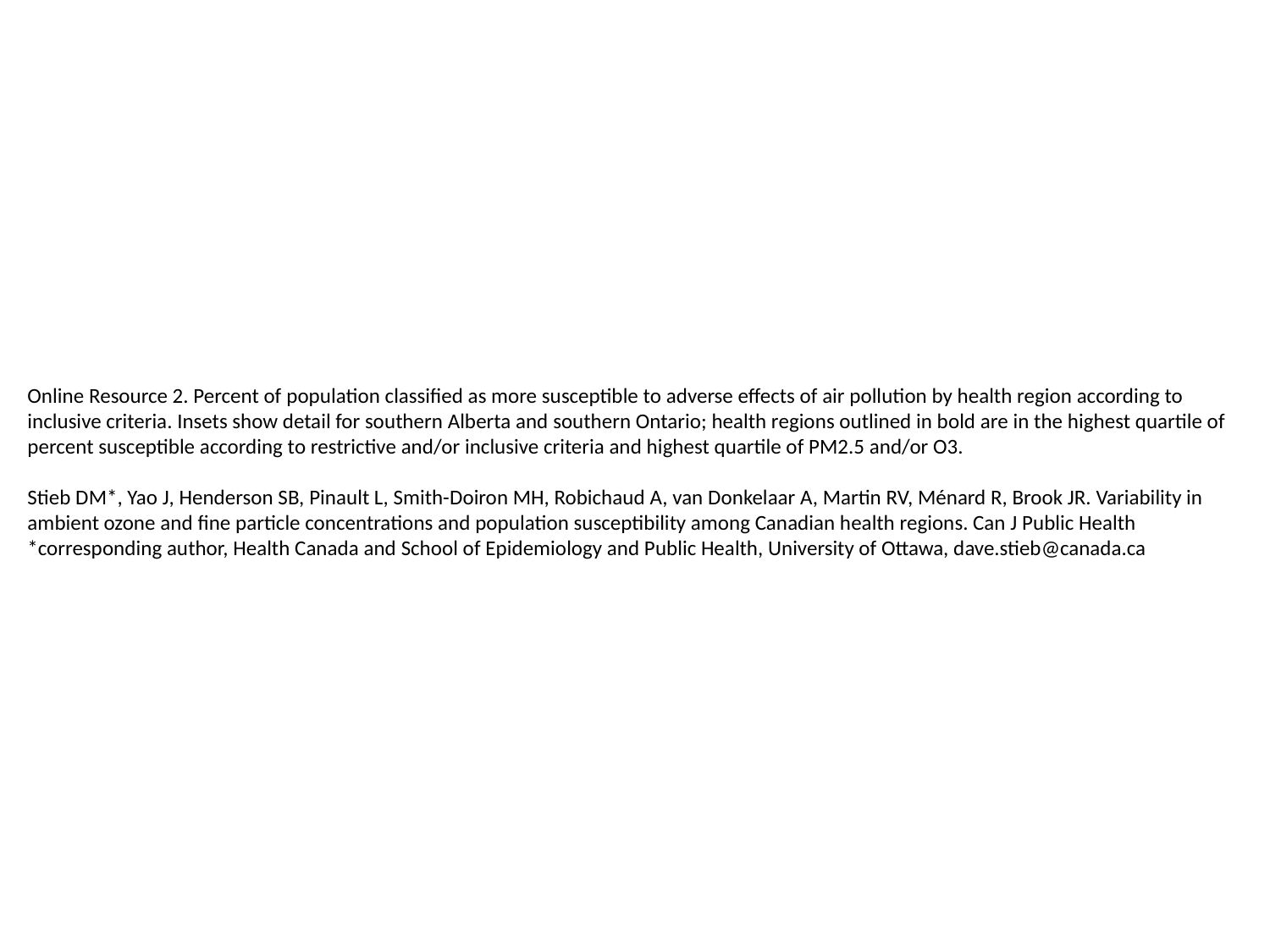

Online Resource 2. Percent of population classified as more susceptible to adverse effects of air pollution by health region according to inclusive criteria. Insets show detail for southern Alberta and southern Ontario; health regions outlined in bold are in the highest quartile of percent susceptible according to restrictive and/or inclusive criteria and highest quartile of PM2.5 and/or O3.
Stieb DM*, Yao J, Henderson SB, Pinault L, Smith-Doiron MH, Robichaud A, van Donkelaar A, Martin RV, Ménard R, Brook JR. Variability in ambient ozone and fine particle concentrations and population susceptibility among Canadian health regions. Can J Public Health
*corresponding author, Health Canada and School of Epidemiology and Public Health, University of Ottawa, dave.stieb@canada.ca

## Slide 2
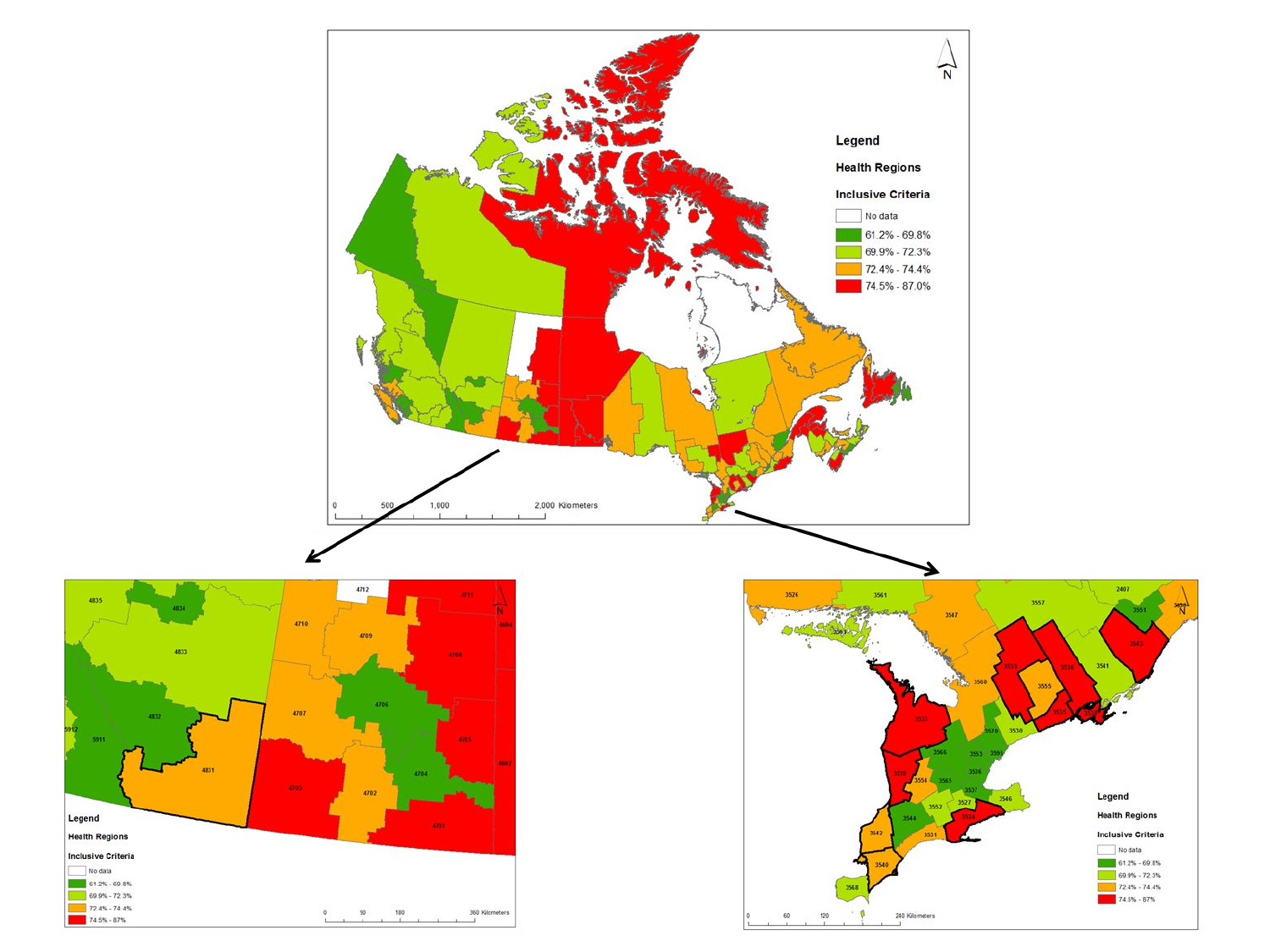

Supplement: Supplementary file 2 — (PPTX 485 kb) [file 41997_2018_169_MOESM2_ESM.pptx]
